# The Ascending Reticular Activating System from Pontine Reticular Formation to the Thalamus in the Human Brain

**DOI:** 10.3389/fnhum.2013.00416

**Published:** 2013-07-25

**Authors:** Sang Seok Yeo, Pyung Hun Chang, Sung Ho Jang

**Affiliations:** ^1^Department of Physical Medicine and Rehabilitation, College of Medicine, Yeungnam UniversityTaegu, South Korea; ^2^Department of Robotic Engineering, Graduate School, Daegu Gyeongbuk Institute of Science & TechnologyTaegu, South Korea

**Keywords:** ascending reticular activating system, diffusion tensor imaging, reticular formation, consciousness

## Abstract

**Introduction:** Action of the ascending reticular activating system (ARAS) on the cerebral cortex is responsible for achievement of consciousness. In this study, we attempted to reconstruct the lower single component of the ARAS from the reticular formation (RF) to the thalamus in the normal human brain using diffusion tensor imaging (DTI).

**Methods:** Twenty six normal healthy subjects were recruited for this study. A 1.5-T scanner was used for scanning of diffusion tensor images, and the lower single component of the ARAS was reconstructed using FMRIB software. We utilized two ROIs for reconstruction of the lower single component of the ARAS: the seed ROI – the RF of the pons at the level of the trigeminal nerve entry zone, the target ROI – the intralaminar nuclei of the thalamus at the level of the commissural plane.

**Results:** The reconstructed ARAS originated from the pontine RF, ascended through the mesencephalic tegmentum just posterior to the red nucleus, and then terminated on the intralaminar nuclei of the thalamus. No significant differences in fractional anisotropy, mean diffusivity, and tract number were observed between hemispheres (*p* > 0.05).

**Conclusion:** We reconstructed the lower single component of the ARAS from the RF to the thalamus in the human brain using DTI. The results of this study might be of value for the diagnosis and prognosis of patients with impaired consciousness.

## Introduction

Consciousness is an arousal and awareness of environment and self, which is achieved through action of the ascending reticular activating system (ARAS) on the brain stem and cerebral cortex (Daube, 1986; Paus, 2000; Zeman, 2001; Gosseries et al., 2011). The ARAS is composed of several neuronal circuits connecting the brainstem to the cortex. These neuronal connections originate mainly in the reticular formation (RF) of the brainstem and project through synaptic relays in the intralaminar nucleus of thalamus to the cerebral cortex (Daube, 1986; Paus, 2000; Zeman, 2001; Afifi and Bergman, 2005; Gosseries et al., 2011). In addition, several brainstem nuclei (locus coeruleus, dorsal raphe, median raphe, pedunculopontine nucleus, parabrachial nucleus), non-specific thalamic nuclei, hypothalamus, and basal forebrain are also included in the ARAS system (Aston-Jones et al., 2001; Parvizi and Damasio, 2003; Fuller et al., 2011). Thorough evaluation of the ARAS is important for diagnosis and management of patients with impaired consciousness, such as patients who are in a vegetative state or those with minimal consciousness (Zeman, 2001; Gosseries et al., 2011).

Conventional brain MRI, functional neuroimaging techniques, electrophysiological methods, and MR spectroscopy have been used in studies of the ARAS in the human brain (Parvizi and Damasio, 2003; Schiff, 2006; Tshibanda et al., 2009, 2010; Gawryluk et al., 2010). However, because the ARAS cannot be clearly discriminated from adjacent neural structures, accurate identification and estimation of the ARAS in the human brain can be problematic when using these methods. In contrast, diffusion tensor imaging (DTI) allows for evaluation of white matter because of its ability to image water diffusion characteristics (Mori et al., 1999). In normal white matter, water molecules have relative freedom of movement parallel to nerve fiber tracts. However, their movements are restricted across tracts, giving rise to diffusion anisotropy of white matter. Accordingly, diffusion anisotropy has been used for evaluation of the extent of fiber change in white matter (Chang et al., 2010; Puig et al., 2010). Several recent studies have attempted to demonstrate the usefulness of DTI for evaluation of lesions in patients with impaired consciousness and connectivity of specific ARAS nuclei in the human brain (Voss et al., 2006; Perlbarg et al., 2009; Tollard et al., 2009; Tshibanda et al., 2009; Fernandez-Espejo et al., 2010, 2011; Newcombe et al., 2010; Edlow et al., 2012). However, little is known about the whole reconstruction and estimation of the ARAS in the human brain (Edlow et al., 2012).

In the current study, using DTI, we attempted to reconstruct the lower single component of the ARAS from the pontine RF to the intralaminar nuclei of the thalamus in the normal human brain.

## Materials and Methods

### Subjects

Twenty six normal healthy subjects (14 males, 12 females; mean age, 31.85 ± 9.80 years; range, 20–50) with no history of neurologic disease were recruited for this study. All subjects participated in this study as volunteers and provided written consent before undergoing DTI scanning. The study was approved by the institutional review board of our hospital.

### Diffusion tensor image

DTI data were acquired using a 6-channel head coil on a 1.5-T Philips Gyroscan Intera (Philips, Best, Netherlands) with single-shot echo-planar imaging. For each of the 32 non-collinear diffusion sensitizing gradients, we acquired 67 contiguous slices parallel to the anterior commissure-posterior commissure line. Imaging parameters were as follows: acquisition matrix = 96 × 96, reconstructed to matrix = 128 × 128, field of view = 221 mm × 221 mm, TR = 10,726 ms, TE = 76 ms, parallel imaging reduction factor (SENSE factor) = 2, EPI factor = 49, and *b* = 1000 s/mm^2^, NEX = 1, and a slice thickness of 2.3 mm (acquired isotropic voxel size 2.3 mm × 2.3 mm × 2.3 mm).

### Probabilistic fiber tracking

Analysis of diffusion-weighted imaging data was performed using the Oxford Centre for Functional Magnetic Resonance Imaging of the Brain (FMRIB) Software Library (FSL; www.fmrib.ox.ac.uk/fsl). Affine multi-scale two-dimensional registration was used for correction of head motion effect and image distortion due to eddy current. Fiber tracking was performed using a probabilistic tractography method based on a multifiber model, and applied in the current study utilizing tractography routines implemented in FMRIB Diffusion (5000 streamline samples, 0.5 mm step lengths, curvature thresholds = 0.2) (Smith et al., 2004). Advantages of probabilistic tractography, which was used in this study, include greater robustness to noise, as well as the ability to detect pathways with sharper angles and to distinguish crossing fibers (Behrens et al., 2007; Winston et al., 2011).

**Figure 1:**
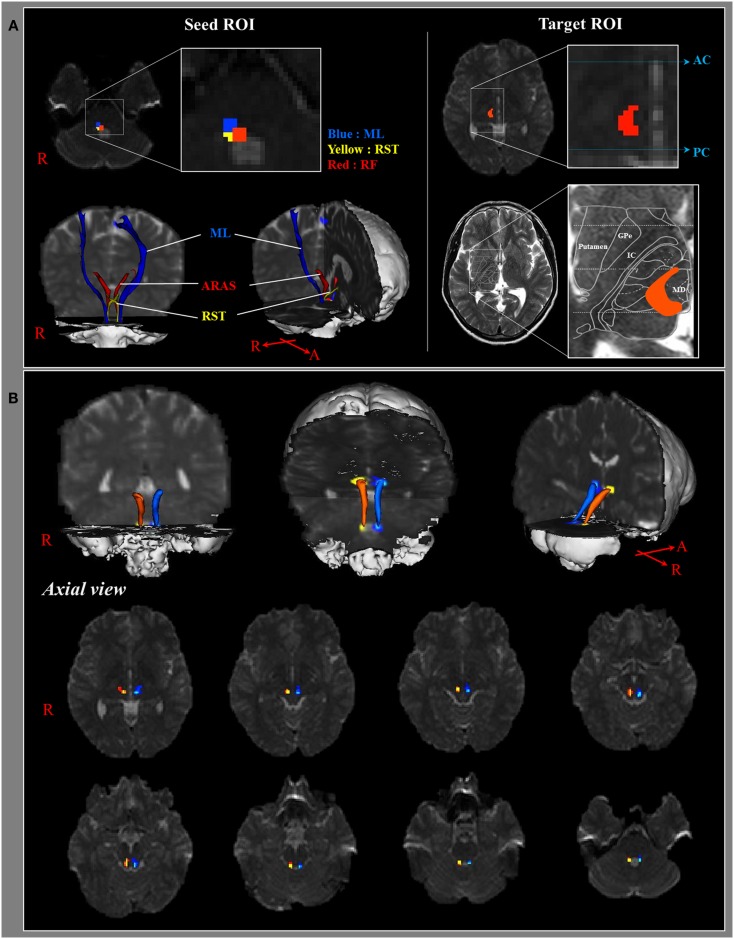
**(A)** Seed regions of interest (ROI) are given on the pontine reticular formation (Red color). The target ROI is given on the intralaminar nuclei of the thalamus at the level of the commissural plane. Boundary of the intralaminar nuclei of the thalamus was defined by reference to the text book of the brain atlas (Morel, 2007). ML, medial lemniscus; RST, rubrospinal tract; RF, reticular formation; AC, anterior commissure; PC, posterior commissure. **(B)** Pathways of the reconstructed ascending reticular activating system are shown at each level of the brain in a normal subject (26-year-old male).

The pathway of the ARAS was determined by selection of fibers passing through seed regions of interest (ROI) and target (termination) ROIs. A seed ROI was placed on the RF of the pons at the level of the trigeminal nerve entry zone (Daube, 1986; Afifi and Bergman, 2005). Analysis of the medial lemniscus and rubrospinal tract was performed in order to confirm the boundary of the RF on the pons (Figure 1A). For analysis of the medial lemniscus, seed ROIs were placed on the anteromedial medulla and the target ROI was placed on the somatosensory cortex (Hong et al., 2010). For analysis of the rubrospinal tract, seed ROIs were placed on the red nucleus and the target ROI was placed on the contralateral dorsolateral region of the medulla (Monakow’s area) (Nathan and Smith, 1982; Kwon et al., 2011). The target ROI was given on the intralaminar nuclei of the thalamus at the level of the commissural plane (Morel, 2007). In defining the intralaminar nuclei of the thalamus, we referred to a brain atlas (Morel, 2007) (Figure 1A). Of 5000 samples generated from the seed voxel, results for contact were visualized at a threshold minimum of 1 streamlined through each voxel for analysis. Values of fractional anisotropy (FA), mean diffusivity (MD), and tract number of the lower single component of ARAS were measured.

### Statistical analysis

SPSS software (v.15.0; SPSS, Chicago, IL, USA) was used for data analysis. Paired *t*-test was used for determination of the difference in values of DTI parameters of the ARAS between the right and left hemispheres. Pearson correlation test was used for determination of correlation between DTI parameters of the ARAS and age. Results were considered significant when the *p* value was <0.05.

## Results

We reconstructed the lower single component of the ARAS between the pontine RF and intralaminar nuclei of the thalamus. The reconstructed component of the ARAS originated from the pontine RF, ascended through the mesencephalic tegmentum just posterior to the red nucleus, and then terminated on the intralaminar nuclei of the thalamus at the level of the commissural plane in all subjects (Figure 1B).

Mean values for FA, MD, and fiber volume of the right ARAS were 0.43 ± 0.05, 0.96 ± 0.13, and 156.96 ± 112.06, respectively, and those of the left ARAS were 0.43 ± 0.04, 0.94 ± 0.13, and 159.73 ± 72.88, respectively. No significant differences in FA, MD, and tract number were observed between the right and left hemispheres (*p* > 0.05) (Table 1). In addition, results of the Pearson correlation test showed no significant age-related changes of FA, MD, and tract number (*p* > 0.05).

**Table 1:** **Diffusion tensor imaging parameters of the ascending reticular activating system**.

	Right hemisphere	Left hemisphere
**ASCENDING RETICULAR ACTIVATING SYSTEM**		
FA	0.43 ± 0.05	0.43 ± 0.04
MD	0.96 ± 0.13	0.94 ± 0.13
Tract number	156.96 ± 112.06	159.73 ± 72.88
**MEDIAL LEMNISCUS**		
FA	0.48 ± 0.04	0.49 ± 0.03
MD	0.83 ± 0.04	0.80 ± 0.07
Tract number	471.50 ± 216.80	378.38 ± 265.52
**RUBROSPINAL TRACT**		
FA	0.47 ± 0.05	0.48 ± 0.03
MD	0.92 ± 0.09	0.91 ± 0.11
Tract number	190.22 ± 109.54	195.93 ± 89.96

*Values represent mean (±SD), FA, Fractional Anisotropy; MD, Mean diffusivity; MD × 10^−3^(mm^2^/s)*.

## Discussion

In the current study, using DTI, we reconstructed one of the main pathways of the ARAS, the lower single component of the ARAS from the RF to the thalamus in normal subjects, although the ARAS consists of additional brainstem nuclei, hypothalamus, basal forebrain, and thalamocortical projections to the cerebral cortex. We selected two ROIs for reconstruction of the lower single component of the ARAS: the seed ROI, which was the RF of the pons at the level of the trigeminal nerve entry zone (Daube, 1986; Afifi and Bergman, 2005), and the target ROI, which included the intralaminar nuclei of the thalamus (the central lateral nuclei, centromedian/parafascicular nuclei, and paracentral nuclei) at the level of the commissural plane (Morel, 2007). The rostral portion of the RF of the brainstem above the trigeminal nerve entry zone is known as the ARAS; in contrast, the caudal portion of the RF is involved in motor function and autonomic function related to cardiac and respiratory function (Daube, 1986). Therefore, we placed the seed ROI in the RF at the level of the trigeminal nerve entry zone. We placed the target ROI in the intralaminar nuclei, which are the main nuclei of the ARAS among the non-specific thalamic nuclei. Therefore, we believe that because we could not include the other thalamic nuclei concerned with the ARAS, the lower single component of the ARAS that was reconstructed in the current study is not the entire lower single component of the ARAS, but the main portion of the entire lower single component of the ARAS. Consequently, the lower single component of the ARAS originated from the pontine RF, ascended through the mesencephalic tegmentum posterior to the red nucleus, and then terminated on the intralaminar nuclei of the thalamus. In addition, values for the FA, MD, and tract numbers of the reconstructed lower single component of the ARAS did not differ significantly between the right and left hemispheres. The tract number is determined by the number of voxels contained within a neural tract (Kwak et al., 2010). The FA value indicates the degree of directionality and integrity of white matter microstructures such as axons, myelin, and microtubules, and the ADC value indicates the magnitude of water diffusion (Assaf and Pasternak, 2008).

Several studies have demonstrated the clinical usefulness of DTI by estimating some areas of the lower single component of the ARAS from the RF to the thalamus in patients with impaired consciousness (Perlbarg et al., 2009; Tollard et al., 2009; Newcombe et al., 2010; Fernandez-Espejo et al., 2011). Tollard et al. (2009) reported on the usefulness of DTI, which was performed at the subacute stage for prediction of outcome in 45 patients with severe TBI (traumatic brain injury) (absence of response to simple orders). In their study, they measured the FA value at several supratentorial and infratentorial areas, including the anterior pons, posterior pons, and midbrain, and demonstrated that the decrease of infratentorial and supratentorial FA, except in the posterior pons, allowed for prediction of unfavorable outcomes 1 year from TBI. Perlbarg et al. (2009), who performed DTI scanning in 30 patients with an absence of response to simple orders following severe TBI, reported a definite decrease in FA measured in the inferior longitudinal fasciculus, midbrain (cerebral peduncle and tegmentum), posterior limb of the internal capsule, and posterior corpus callosum in the unfavorable outcome group. Newcombe et al. (2010) used DTI for characterization of the extent and location of white matter loss in patients who were in a vegetative state secondary to TBI (seven patients) and patients with ischemic-hypoxic injury (five patients). Abnormalities in the supratentorial areas were observed in both groups; in contrast, abnormalities of the brainstem were observed only in the TBI group. Fernandez-Espejo et al. (2011) used DTI in differentiation of the neuropathology of 25 vegetative and minimally conscious patients. They concluded that minimally conscious patients and those in a vegetative state differed in subcortical white matter and thalamic regions, but appeared not to differ in the brainstem. In a recent study using high angular resolution diffusion imaging, Edlow et al. (2012) reported on neuroanatomical connectivity of the ARAS in the human brain, both *in vivo* and *ex vivo*. They demonstrated that the connectivities of specific ARAS nuclei were implicated in arousal, and those of thalamic nuclei were implicated in modulation of arousal.

In conclusion, using DTI, we reconstructed the lower single component of the ARAS from the RF to the thalamus in the human brain. We believe that the methodology used and the results of this study may be helpful to researchers studying the ARAS in the human brain. However, one of the limitations of this study is that we were not able to fully elucidate the entire ARAS system because we did not include other thalamic and brainstem nuclei in our analysis which are also involved in the ARAS. Further studies on the clinical usefulness of our findings as well as studies on the projections of the ARAS from the thalamus to the cerebral cortex are needed.

## Conflict of Interest Statement

The authors declare that the research was conducted in the absence of any commercial or financial relationships that could be construed as a potential conflict of interest.

## References

[B1] Afifi A. K. Bergman R. A. (2005). Functional Neuroanatomy: Text and Atlas. New York: Lange Medical Books/McGraw-Hill

[B2] Assaf Y. Pasternak O. (2008). Diffusion tensor imaging (DTI)-based white matter mapping in brain research: a review. J. Mol. Neurosci. 34, 51–61 10.1007/s12031-007-0029-0 18157658

[B3] Aston-Jones G. Chen S. Zhu Y. Oshinsky M. L. (2001). A neural circuit for circadian regulation of arousal. Nat. Neurosci. 4, 732–738 10.1038/89522 11426230

[B4] Behrens T. E. Berg H. J. Jbabdi S. Rushworth M. F. Woolrich M. W. (2007). Probabilistic diffusion tractography with multiple fibre orientations. Neuroimage 34, 144–155 10.1016/j.neuroimage.2006.09.018 17070705 PMC7116582

[B5] Chang M. C. Kim S. H. Kim O. L. Bai D. S. Jang S. H. (2010). The relation between fornix injury and memory impairment in patients with diffuse axonal injury: a diffusion tensor imaging study. NeuroRehabilitation 26, 347–353 10.3233/NRE-2010-0572 20555158

[B6] Daube J. R. (1986). Medical Neurosciences: An Approach to Anatomy, Pathology, and Physiology by Systems and Levels. Boston: Little, Brown and Co

[B7] Edlow B. L. Takahashi E. Wu O. Benner T. Dai G. Bu L. (2012). Neuroanatomic connectivity of the human ascending arousal system critical to consciousness and its disorders. J. Neuropathol. Exp. Neurol. 71, 531–546 10.1097/NEN.0b013e3182588293 22592840 PMC3387430

[B8] Fernandez-Espejo D. Bekinschtein T. Monti M. M. Pickard J. D. Junque C. Coleman M. R. (2011). Diffusion weighted imaging distinguishes the vegetative state from the minimally conscious state. Neuroimage 54, 103–112 10.1016/j.neuroimage.2010.08.035 20728553

[B9] Fernandez-Espejo D. Junque C. Cruse D. Bernabeu M. Roig-Rovira T. Fabregas N. (2010). Combination of diffusion tensor and functional magnetic resonance imaging during recovery from the vegetative state. BMC Neurol. 10:77 10.1186/1471-2377-10-77 20815871 PMC2941677

[B10] Fuller P. M. Sherman D. Pedersen N. P. Saper C. B. Lu J. (2011). Reassessment of the structural basis of the ascending arousal system. J. Comp. Neurol. 519, 933–956 10.1002/cne.22559 21280045 PMC3119596

[B11] Gawryluk J. R. D’Arcy R. C. Connolly J. F. Weaver D. F. (2010). Improving the clinical assessment of consciousness with advances in electrophysiological and neuroimaging techniques. BMC Neurol. 10:11 10.1186/1471-2377-10-11 20113490 PMC2828440

[B12] Gosseries O. Bruno M. A. Chatelle C. Vanhaudenhuyse A. Schnakers C. Soddu A. (2011). Disorders of consciousness: what’s in a name? NeuroRehabilitation 28, 3–14 21335671 10.3233/NRE-2011-0625

[B13] Hong J. H. Son S. M. Jang S. H. (2010). Identification of spinothalamic tract and its related thalamocortical fibers in human brain. Neurosci. Lett. 468, 102–105 10.1016/j.neulet.2009.10.075 19879333

[B14] Kwak S. Y. Yeo S. S. Choi B. Y. Chang C. H. Jang S. H. (2010). Corticospinal tract change in the unaffected hemisphere at the early stage of intracerebral hemorrhage: a diffusion tensor tractography study. Eur. Neurol. 63, 149–153 10.1159/000281108 20134168

[B15] Kwon H. G. Hong J. H. Hong C. P. Lee D. H. Ahn S. H. Jang S. H. (2011). Dentatorubrothalamic tract in human brain: diffusion tensor tractography study. Neuroradiology 53, 787–791 10.1007/s00234-011-0878-7 21547376

[B16] Morel A. (2007). Stereotactic Atlas of the Human Thalamus and Basal Ganglia. New York: Informa Healthcare

[B17] Mori S. Crain B. J. Chacko V. P. van Zijl P. C. (1999). Three-dimensional tracking of axonal projections in the brain by magnetic resonance imaging. Ann. Neurol. 45, 265–269 10.1002/1531-8249(199902)45:2<265::AID-ANA21>3.0.CO;2-3 9989633

[B18] Nathan P. W. Smith M. C. (1982). The rubrospinal and central tegmental tracts in man. Brain 105, 223–269 10.1093/brain/105.2.223 7082990

[B19] Newcombe V. F. Williams G. B. Scoffings D. Cross J. Carpenter T. A. Pickard J. D. (2010). Aetiological differences in neuroanatomy of the vegetative state: insights from diffusion tensor imaging and functional implications. J. Neurol. Neurosurg. Psychiatry 81, 552–561 10.1136/jnnp.2009.196246 20460593

[B20] Parvizi J. Damasio A. R. (2003). Neuroanatomical correlates of brainstem coma. Brain 126, 1524–1536 10.1093/brain/awg166 12805123

[B21] Paus T. (2000). Functional anatomy of arousal and attention systems in the human brain. Prog. Brain Res. 126, 65–77 10.1016/S0079-6123(00)26007-X 11105640

[B22] Perlbarg V. Puybasset L. Tollard E. Lehericy S. Benali H. Galanaud D. (2009). Relation between brain lesion location and clinical outcome in patients with severe traumatic brain injury: a diffusion tensor imaging study using voxel-based approaches. Hum. Brain Mapp. 30, 3924–3933 10.1002/hbm.20817 19507154 PMC6870894

[B23] Puig J. Pedraza S. Blasco G. Daunis I. E. J. Prats A. Prados F. (2010). Wallerian degeneration in the corticospinal tract evaluated by diffusion tensor imaging correlates with motor deficit 30 days after middle cerebral artery ischemic stroke. AJNR Am. J. Neuroradiol. 31, 1324–1330 10.3174/ajnr.A2038 20299434 PMC7965455

[B24] Schiff N. D. (2006). Multimodal neuroimaging approaches to disorders of consciousness. J. Head Trauma Rehabil. 21, 388–397 10.1097/00001199-200609000-00003 16983224

[B25] Smith S. M. Jenkinson M. Woolrich M. W. Beckmann C. F. Behrens T. E. Johansen-Berg H. (2004). Advances in functional and structural MR image analysis and implementation as FSL. Neuroimage 23(Suppl. 1), S208–S219 10.1016/j.neuroimage.2004.07.051 15501092

[B26] Tollard E. Galanaud D. Perlbarg V. Sanchez-Pena P. Le Fur Y. Abdennour L. (2009). Experience of diffusion tensor imaging and 1H spectroscopy for outcome prediction in severe traumatic brain injury: preliminary results. Crit. Care Med. 37, 1448–1455 10.1097/CCM.0b013e31819cf050 19242330

[B27] Tshibanda L. Vanhaudenhuyse A. Boly M. Soddu A. Bruno M. A. Moonen G. (2010). Neuroimaging after coma. Neuroradiology 52, 15–24 10.1007/s00234-009-0614-8 19862509

[B28] Tshibanda L. Vanhaudenhuyse A. Galanaud D. Boly M. Laureys S. Puybasset L. (2009). Magnetic resonance spectroscopy and diffusion tensor imaging in coma survivors: promises and pitfalls. Prog. Brain Res. 177, 215–229 10.1016/S0079-6123(09)17715-4 19818904

[B29] Voss H. U. Uluc A. M. Dyke J. P. Watts R. Kobylarz E. J. McCandliss B. D. (2006). Possible axonal regrowth in late recovery from the minimally conscious state. J. Clin. Invest. 116, 2005–2011 10.1172/JCI27021 16823492 PMC1483160

[B30] Winston G. P. Mancini L. Stretton J. Ashmore J. Symms M. R. Duncan J. S. (2011). Diffusion tensor imaging tractography of the optic radiation for epilepsy surgical planning: a comparison of two methods. Epilepsy Res. 97, 124–132 10.1016/j.eplepsyres.2011.07.019 21885257 PMC3223565

[B31] Zeman A. (2001). Consciousness. Brain 124, 1263–1289 10.1093/brain/124.7.1263 11408323

